# The systemic immune-inflammation index is significantly associated with the severity of silicosis: a 9-year retrospective study in Beijing

**DOI:** 10.3389/fmed.2024.1351589

**Published:** 2024-02-07

**Authors:** Han-Yu-Jie Kang, Si-Yu Cao, Shuai Shao, Li-Rong Liang, Zhao-Hui Tong

**Affiliations:** ^1^Department of Respiratory and Critical Care Medicine, Beijing Institute of Respiratory Medicine, Beijing Chao-Yang Hospital, Capital Medical University, Beijing, China; ^2^Department of Clinical Epidemiology, Beijing Institute of Respiratory Medicine and Beijing Chao-Yang Hospital, Capital Medical University, Beijing, China

**Keywords:** occupational disease, silicosis, severity, systemic immune-inflammation index, lung function

## Abstract

**Background:**

Silicosis shows an increasing trend with the development of new industries. However, the potential biomarkers for predicting the disease severity are lacking. A novel inflammatory marker, the systemic immune-inflammation Index (SII), has not been studied in silicosis.

**Methods:**

In this retrospective study, we used data from a big database platform of a tertiary general hospital in Beijing, which was established based on the electronic medical records of the hospital. The clinical data of adult patients diagnosed with silicosis at the Department of Occupational Medicine and Toxicology from 2013 to 2022 were collected. The data extracted from the database were in de-identified form. Only patients with a first diagnosis of silicosis and without conditions that might affect the parameters of routine blood tests were included in the analysis. Analyses were performed to assess the relationship between SII and the advanced stage of silicosis.

**Results:**

A total of 246 participants were included in the study. Most of the patients were exposed to silica particles during excavation and digging (*n* = 149, 60.6%). SII level was significantly higher in patients with advanced stages of silicosis. A multivariate logistic regression analysis revealed that a higher SII level was associated with the advanced stage of silicosis [odds ratio (OR) = 1.002; 95% confidence interval (CI): 1.000–1.003, *p* < 0.001] after adjusting for all covariates. The best cutoff value of SII was 444.1. The results of the subgroup analysis also showed a significant correlation between SII level over 444.1 and the advanced stage of silicosis in groups stratified by gender, history of smoking, and duration of silica exposure. Moreover, our results showed a significant but weak negative correlation between the level of SII and some lung function parameters in silicosis.

**Conclusion:**

Higher SII is associated with the advanced stage of silicosis and impaired lung function. More long-term, large-scale studies are needed to confirm these findings.

## Introduction

Silicosis is a disease characterized by progressive pulmonary fibrosis caused by long-term inhalation of free silica dust in many industries (1). The formation of silicotic nodules and diffuse interstitial pulmonary fibrosis characterizes it (1). Silicosis is an occupational disease with relatively high morbidity and mortality (2). Globally, 2.65 million cases of silicosis were reported in 2019 (3). Silicosis is rising with new industries, such as artificial stone mesa manufacturing and jewelry polishing (2). There are still no effective treatments, and lung transplantation is often needed in the late stage (4). Therefore, it is essential to explore markers associated with the severity of the disease to help easily and quickly identify advanced-stage silicosis, which may be helpful to guide clinical management. It has been shown that the incidence of silicosis may be influenced by many factors, such as duration of dust exposure, cumulative total dust exposure, and genetic variants (5, 6). However, markers that may be associated with the severity of silicosis are very limited.

The systemic immune-inflammation index (SII) is an index that represents the body’s systemic immune-inflammatory response based on the peripheral neutrophil, platelet, and lymphocyte counts (7).

Given the ease of access to these routine blood indicators, SII has received much attention in recent years. Many studies have explored the correlation between SII and the prognosis of some serious diseases, such as cancer, sepsis, and cardiovascular disease (8–10). According to a recent study, SII greater than 500 is a marker of pulmonary interstitial involvement in connective diseases (11). There is a close relationship between immunity and inflammation with the development of silicosis (2). Persistent inflammation and progressive worsening of lung fibrosis are characteristics of silicosis (12). However, little is known about the association between SII and the severity of silicosis.

Therefore, the focus of this study was to assess the association between SII and the severity of silicosis and further explore the predictive value of SII for the severity of silicosis, with the aim of helping the management of silicosis.

## Methods

### Research design and study population

This was a retrospective study using the big data platform of Beijing Chao-Yang Hospital, which was based on electronic medical records. We included patients diagnosed with silicosis at the Department of Occupational Medicine and Toxicology, Beijing Chao-Yang Hospital, between 2013 and 2022. Silicosis was diagnosed through multidisciplinary discussions on the basis of the occupational history of exposure to silica dust and the radiological criteria based on the International Labor Organization classification (13). If a patient had more than one hospitalization, only the one for the first diagnosis of silicosis was included for analysis. Patients with occupational lung diseases other than silicosis were not included. We also excluded patients who had diseases (pneumonia, tuberculosis, other infectious diseases, lung cancer) or were using drugs (immunosuppressive drugs) that could affect the parameters of routine blood tests. In addition, patients lacking information on silicosis staging, the duration of silica exposure, smoking history, and routine blood tests were excluded.

A total of 246 patients were included in the analysis. The study was approved by the Research Ethics Board of Beijing Chao-Yang Hospital (2023-ke-357). Data in the big data platform were de-identified. The patient’s personal information was not identifiable, so informed consent was not required. The study adhered to the STROBE Guidelines, and the checklist is presented in Supplementary Table S1.

### Classification of the stage of silicosis by chest radiograph

Silicosis was classified into three stages based on the International Labor Organization staging classification system (13) by multidisciplinary discussions. In short, posterior chest radiographs showed that each lung field was separated into three zones, namely upper, middle, and lower. The patients were classified as stage I when the distribution affected two or more zones, the greatest density of tiny opacities was ≥1/0, and pleural plaques were seen. The patients were classified as stage II when the distribution covered more than four zones and the highest density of small opacities was ≥2/1. The patients were classified as stage III when the biggest opacity measured ≥20 × 10 mm in diameter, or when the distribution affected four or more zones with aggregation of tiny or large opacities and the highest density of small opacities was ≥3/2. The advanced stage of silicosis was defined as stages II and III.

### Data collection

Clinical records of the patients during hospitalization were obtained from the database. Specifically, demographic characteristics, including age, gender, duration of silica exposure, history of smoking, history of alcohol intake, and comorbidities, were collected. In addition, we extracted data on the patients’ vital signs and symptoms on admission, silicosis stage, laboratory tests, lung function parameters, and outcome indicators (length of hospital stay and hospital mortality) from the database.

### Calculation of the SII index

We collected neutrophil, platelet, and lymphocyte counts from admission routine blood tests to assess the SII index. The following formula was used to calculate the SII index: (neutrophil count × platelet count)/lymphocyte count (7, 14).

### Covariates

The study adjusted for covariates that could affect the correlation between SII and the severity of silicosis. In detail, many variables, including baseline characteristics (such as age, gender, duration of silica exposure, history of smoking, and history of smoking ≥10 pack-years), and laboratory tests [such as neutrophil count, lymphocyte count, platelet count, and C-reactive protein (CRP)], were considered.

### Statistical analysis

For continuous variables, normally distributed data are presented as the mean ± standard deviation, and non-normally distributed data are presented as the median with an interquartile range. For categorical variables, data are presented as frequencies (percentages). Student’s *t*-test or Mann–Whitney U test was used for comparing continuous variables. Categorical variables were compared by the Chi-square test or Fisher’s exact test.

The receiver operating characteristic (ROC) curve was used to access the optimal cutoff value of the SII according to the Youden index. Before multivariate analysis, bivariate analyses were performed. The variables with a *p*-value lower than 0.05 and other covariates that could be associated with advanced silicosis were included in the multivariate logistic regression model for adjustment. Then, multivariate logistic regression analysis was performed to investigate the association between SII and the advanced stage of silicosis. Sensitivity analyses were performed by subgroup analyses, which were further used to explore the association between SII and severe silicosis in different subgroups. The subgroups were stratified by gender, smoking history, and silica exposure duration. The interaction test assessed the heterogeneity of the association between the subgroups. ROC curves were used to judge the sensitivity of markers to identify the advanced stage of silicosis. The Pearson r test or Spearman test was used to explore the correlation between the variables. All statistical analyses were performed using SPSS version 26.0 (SPSS Inc., Chicago, IL, United States) and the statistical software package R. A *p* < 0.05 was considered statistically significant.

## Results

### Patient characteristics

**Figure 1:**
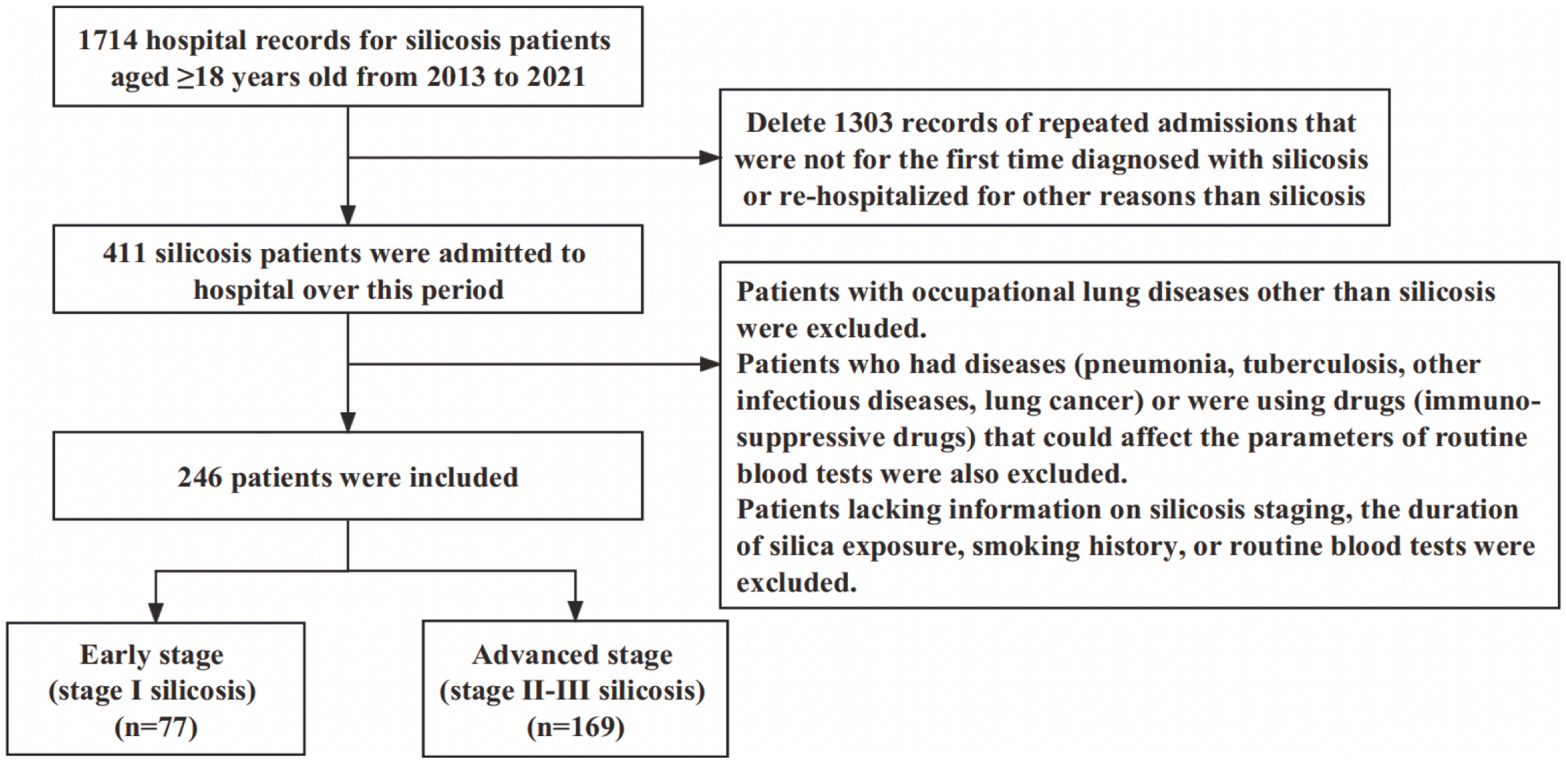
Flowchart of the participants selection.

After the selection process presented in Figure 1, a total of 246 eligible patients were included in the study. More than 50% of the patients were workers with an occupational history of exposure to silica dust during excavation and digging (149, 60.6%), followed by polishing and buffing (53, 21.5%), handling raw materials (24, 9.8%), and rock blasting and sand blasting (20, 8.1%). The median age was 61 years (IQR, 54–70), and the median duration of silica exposure was 20 years (IQR, 12–25). Most of the patients were male (84.1%), and only a small portion of the patients in this study were female (15.9%). Among these patients, 106 (43.1%) had a smoking history. A significant smoking history of ≥10 pack-years was reported by 77 (31.3%) patients. Only 57 (23.2%) patients had a history of alcohol intake. The main symptoms of these patients on admission were coughing sputum (*n* = 159, 64.6%), cough (*n* = 131, 53.3%), and dyspnea (*n* = 106, 43.1%). Although coughing sputum was the most obvious symptom, it was not caused by a chest infection, given that we excluded patients with infectious diseases. The symptom may have been related to silicosis itself, possibly due to silica dust irritation. The median length of hospital stay was 11 days (IQR, 8–13 days), and the hospital mortality was only 2.0% (5/246). Advanced stage silicosis (stage II-III silicosis) was present in 169 (68.7%) subjects. The characteristics of the 246 patients are summarized in Table 1.

**Table 1:** Characteristics of patients with silicosis according to early (stage I silicosis) versus advanced (stage II-III silicosis) stage.

	Overall (*n* = 246)	Stage I silicosis (*n* = 77)	Stage II-III silicosis (*n* = 169)	*p*-value
**Baseline characteristics**				
Age, years	61.0 (54.0–70.0)	63.0 (58.5–69.0)	61.0 (52.5–71.5)	0.163
Male, n (%)	207 (84.1%)	58 (75.3%)	149 (88.2%)	0.011
Duration of exposure (years)	20.0 (12.0–25.0)	17.0 (11.0–23.5)	21.0 (12.5–26.0)	0.020
History of smoking, n (%)	106 (43.1%)	26 (33.8%)	80 (47.3%)	0.046
History of smoking ≥10 pack-years	77 (31.3%)	15 (19.5%)	62 (36.7%)	0.007
History of alcohol intake, n (%)	57 (23.2%)	15 (19.5%)	42 (24.9%)	0.354
**Comorbidities, *n* (%)**				
Diabetes	35 (14.2%)	10 (13%)	25 (14.8%)	0.707
Hyperlipidemia	6 (2.4%)	3 (3.9%)	3 (1.8%)	0.317
Hypertension	81 (32.9%)	19 (24.7%)	62 (36.7%)	0.063
Coronary heart disease	2 (0.8%)	1 (1.3%)	1 (0.6%)	0.567
Stroke	7 (2.8%)	4 (5.2%)	3 (1.8%)	0.135
**On hospital admission**				
Heart rate, bpm	80 (76–90)	80 (72–84)	80 (76–93)	0.068
Systolic blood pressure, mmHg	125 (120–138)	121 (118–130)	125 (120–140)	0.064
Diastolic blood pressure, mmHg	75 (70–80)	73 (70–80)	77 (70–80)	0.530
**Symptoms**				
Chest pain, n (%)	7 (2.8%)	2 (2.6%)	5 (3.0%)	0.618
Chest distress, n (%)	26 (10.6%)	6 (7.8%)	20 (11.8%)	0.339
Coughing sputum, n (%)	159 (64.6%)	53 (68.8%)	106 (62.7%)	0.353
Cough, n (%)	131 (53.3%)	38 (49.4%)	93 (55.0%)	0.408
Hemoptysis, n (%)	12 (4.9%)	1 (1.3%)	11 (6.5%)	0.067
Dyspnea, n (%)	106 (43.1%)	35 (45.5%)	71 (42.0%)	0.613
Shortness of breath, n (%)	43 (17.5%)	9 (11.7%)	34 (20.1%)	0.106
**Laboratory tests**				
White blood cell, 10^9^/L	6.28 (5.19–7.70)	6.06 (4.95–7.14)	6.39 (5.24–7.82)	0.126
Neutrophil, 10^9^/L	4.03 (3.18–5.01)	3.81 (2.86–4.57)	4.23 (3.35–5.44)	0.012
Lymphocyte, 10^9^/L	1.54 (1.16–1.99)	1.70 (1.38–2.14)	1.50 (1.06–1.86)	0.002
Monocyte, 10^9^/L	0.43 (0.34–0.55)	0.42 (0.30–0.51)	0.44 (0.34–0.56)	0.097
Platelet, 10^9^/L	214 (175–261)	203 (173–247)	222 (176–270)	0.042
Hemoglobin, g/L	133 (118–145)	134 (125–145)	133 (115–146)	0.296
CRP, mg/L	0.74 (0.28–1.75)	0.38 (0.25–1.32)	0.84 (0.33–2.23)	0.134
SII, 10^9^/L	529.7 (396.1–927.6)	415.2 (289.5–676.4)	569.8 (438.1–1060.5)	<0.001
**Outcome**				
Length of hospital stay, days	11.0 (8.0–13.0)	10.0 (8.0–13.0)	11.0 (8.0–13.5)	0.127
Hospital mortality, n (%)	5 (2.0%)	1 (1.3%)	4 (2.4%)	0.501

The enumeration data indicators were described by frequency/percentage and compared by the Chi-square or Fisher’ s exact test. The continuous variables with normal distribution were described by mean ± standard deviation, and median and interquartile ranges were used to describe the non-normal distribution variables. Student’ s *t*-test compared variables with normal distribution, and variables with non-normal distribution were compared by Mann–Whitney test. Significance was set as *p* < 0.05.

Compared with the early stage of silicosis (stage I silicosis), the patients in the advanced stage of silicosis (stage II-III silicosis) had a longer duration of silica exposure (*p* = 0.020). More male genders were also found in the group of advanced stage of silicosis (*p* = 0.011). There were 26 subjects (33.8%) in stage I silicosis (early stage) and 80 subjects (47.3%) in stage II-III silicosis (advanced stage) with a history of smoking (*p* = 0.046). Especially patients with a history of smoking ≥10 pack-years were significantly more frequent (*p* = 0.007) among patients with stage II-III silicosis (36.7%) compared to those with stage I silicosis (19.5%). The neutrophil counts (*p* = 0.012) and platelet counts (*p* = 0.042) were higher in patients with advanced stages of silicosis, whereas lymphocyte count was lower (*p* = 0.002). SII level was significantly higher in patients in the advanced stages of silicosis relative to those in the early stages (*p* < 0.001). More details of the comparison between the patients with stage I silicosis and stage II-III silicosis are shown in Table 1.

### SII and the severity of silicosis

We created a multivariate logistic regression model to explore the factors that may be associated with the advanced stage of silicosis. Baseline characteristics associated with the advanced stage of silicosis by bivariate analysis and other potential confounding factors were included in the multivariate analysis. The details of adjusting for confounding factors are presented in Table 2. In the multivariate logistic regression, the SII level (OR = 1.002, 95% CI: 1.000–1.003, *p* < 0.001), male (OR = 3.909, 95% CI: 1.319–11.581, *p* = 0.014), duration of silica exposure (OR = 1.038, 95% CI: 1.003–1.075, *p* = 0.031) and history of smoking ≥10 pack-years (OR = 2.112, 95% CI: 1.075–4.147, *p* = 0.030) were significantly associated with the advanced stage of silicosis.

**Table 2:** The logistic regression model revealed the association between the systemic immune-inflammation index and the stage of silicosis.*

Variables	OR (95% CI)	*p*
SII	1.002 (1.000–1.003)	<0.001
Male	3.909 (1.319–11.581)	0.014
Duration of exposure (years)	1.038 (1.003–1.075)	0.031
History of smoking ≥10 pack-years	2.112 (1.075–4.147)	0.030
History of smoking	0.559 (0.139–2.246)	0.413
Age	0.980 (0.953–1.007)	0.144
Neutrophil counts	0.612 (0.329–1.139)	0.122
Lymphocyte counts	0.704 (0.136–3.652)	0.676
Platelet counts	1.003 (0.989–1.017)	0.668
CRP	0.938 (0.733–1.200)	0.610

OR, odds ratio; CI, confidence interval.

*Whole Model Test < 0.001.

*n = 246.

*The model adjusted for age, gender, duration of silica exposure, history of smoking, history of smoking ≥10 pack-years, neutrophil counts, lymphocyte counts, platelet counts, and CRP.

To further explore the relationship between the SII index and the advanced stage of silicosis, we performed ROC curve analysis to determine the optimal cutoff value of the SII. The results revealed that the optimal cutoff value of SII was 444.1 for the advanced stage of silicosis. Thus, we classified the patients with an SII value greater than 444.1 into the high SII group, while all other patients were defined as the low SII group. Similarly, a multivariate logistic regression model was used. As shown in Table 3, the multivariate analysis showed that high SII levels were significantly associated with the advanced stage of silicosis (OR = 5.110, 95% CI 2.807–9.302, *p* < 0.001). In addition, a history of smoking ≥10 pack-years (OR = 2.249, 95% CI 1.125–4.496, *p* = 0.022) and a longer duration of silica exposure (OR = 1.044, 95% CI 1.007–1.082, *p* = 0.018) were also associated with more severe silicosis (stage II-III silicosis) in the study.

**Table 3:** The logistic regression model revealed the association between higher SII (SII ≥ 444.1) and the advanced stage (stage II-III silicosis) of silicosis.*

Variables	OR (95% CI)	*p*
SII ≥444.1	5.110 (2.807–9.302)	<0.001
Duration of exposure (years)	1.044 (1.007–1.082)	0.018
History of smoking ≥10 pack-years	2.249 (1.125–4.496)	0.022
Male	3.201 (0.882–11.626)	0.077
Age	0.976 (0.947–1.006)	0.110
History of smoking	0.766 (0.294–1.998)	0.586
Neutrophil counts	1.046 (0.823–1.330)	0.713
Lymphocyte counts	0.613 (0.321–1.172)	0.139
Platelet counts	1.002 (0.997–1.008)	0.401
CRP	0.986 (0.793–1.225)	0.896

OR, odds ratio; CI, confidence interval.

*Whole Model Test < 0.001.

*n = 246.

*The model adjusted for age, gender, duration of silica exposure, history of smoking, history of smoking ≥10 pack-years, neutrophil counts, lymphocyte counts, platelet counts, and CRP.

### Subgroup analysis

In Table 4, subgroup analyses based on the factors that may influence the severity of silicosis in the above regression models were performed to explore the association between the SII and severe silicosis. The results of the subgroup analysis indicated that the positive correlation between SII and the advanced stage of silicosis was significant in all of the subgroups stratified by gender, history of smoking, and duration of silica exposure. The interaction test suggested no significant effects of different genders, history of smoking, and duration of silica exposure on the positive relationship between the SII and the advanced stage of silicosis (*p* for interaction >0.05). More information about the subgroup analysis of the confounding factors is shown in Supplementary Tables S2–S7.

**Table 4:** Subgroup analysis of the association between higher SII and advanced stage (stage II-III silicosis) of silicosis.*

Subgroup	No of participants	OR (95% CI)	*p*	*p* for interaction
**Gender**				0.753
Male	143	4.793 (2.435, 9.435)	<0.001	
Female	16	5.625 (1.359, 23.274)	0.017	
**History of smoking**				0.536
≥10 pack-years	56	4.308 (1.318, 14.081)	0.016	
<10 pack-years	103	5.571 (2.763, 11.232)	<0.001	
**Duration of exposure (years)**				0.759
>20 years	77	6.469 (2.493, 16.786)	<0.001	
≤20 years	82	4.747 (2.121, 10.626)	<0.001	

*The model adjusted for age, gender, duration of silica exposure, history of smoking, history of smoking ≥10 pack-years, neutrophil counts, lymphocyte counts, platelet counts, and CRP.

### Correlations between SII and pulmonary function test

**Figure 2:**
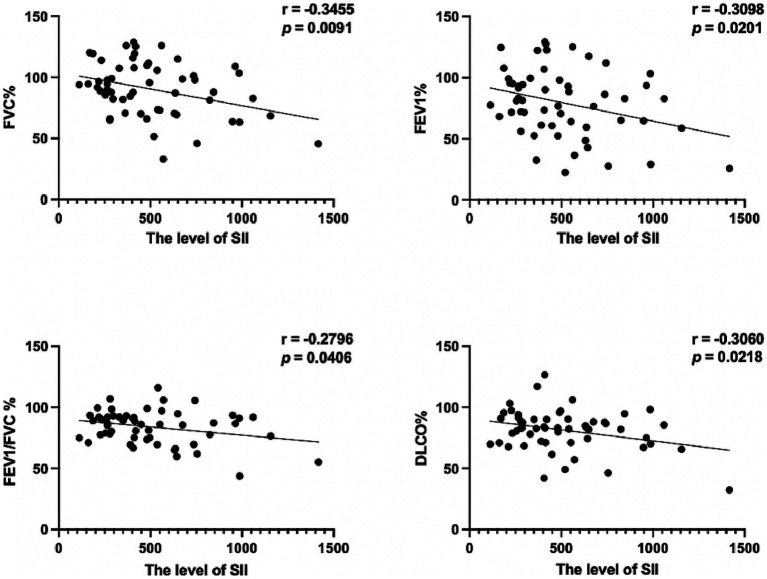
Correlations between SII and pulmonary function test.

Of these patients, a total of 56 underwent pulmonary function tests. We found that lung function parameters, such as FVC (% pred), FEV1 (% pred), FEV1/FVC %, and DLCO (% pred), were significantly different between the advanced stages and early stages of silicosis. The results are shown in Table 5. These parameters negatively correlated with the level of SII (Figure 2). The results revealed the relatively poor pulmonary function in silicosis, which is possibly related to higher SII levels. Given the obvious difference in lung function parameters between the advanced and early stages of silicosis, the association may be more due to the higher SII levels in more advanced stages of silicosis. Hence, the findings again suggested a correlation between the SII and disease severity.

**Table 5:** Lung functions of patients with silicosis according to early (stage I silicosis) versus advanced (stage II-III silicosis) stage.

	Overall (*n* = 56)	Stage I silicosis (*n* = 28)	Stage II–III silicosis (*n* = 28)	*p*-value
FVC (% pred)	90.2 ± 22.3	97.0 ± 19.0	83.3 ± 19.0	0.020
FEV1 (% pred)	79.4 ± 27.8	89.8 ± 23.2	69.0 ± 28.5	0.004
FEV1/FVC %	83.8 ± 13.8	88.0 ± 8.5	79.5 ± 16.7	0.023
DLCO (% pred)	81.4 ± 16.8	87.3 ± 11.3	75.4 ± 19.3	0.007

### Predictive power of high SII levels for the severity of silicosis

**Figure 3:**
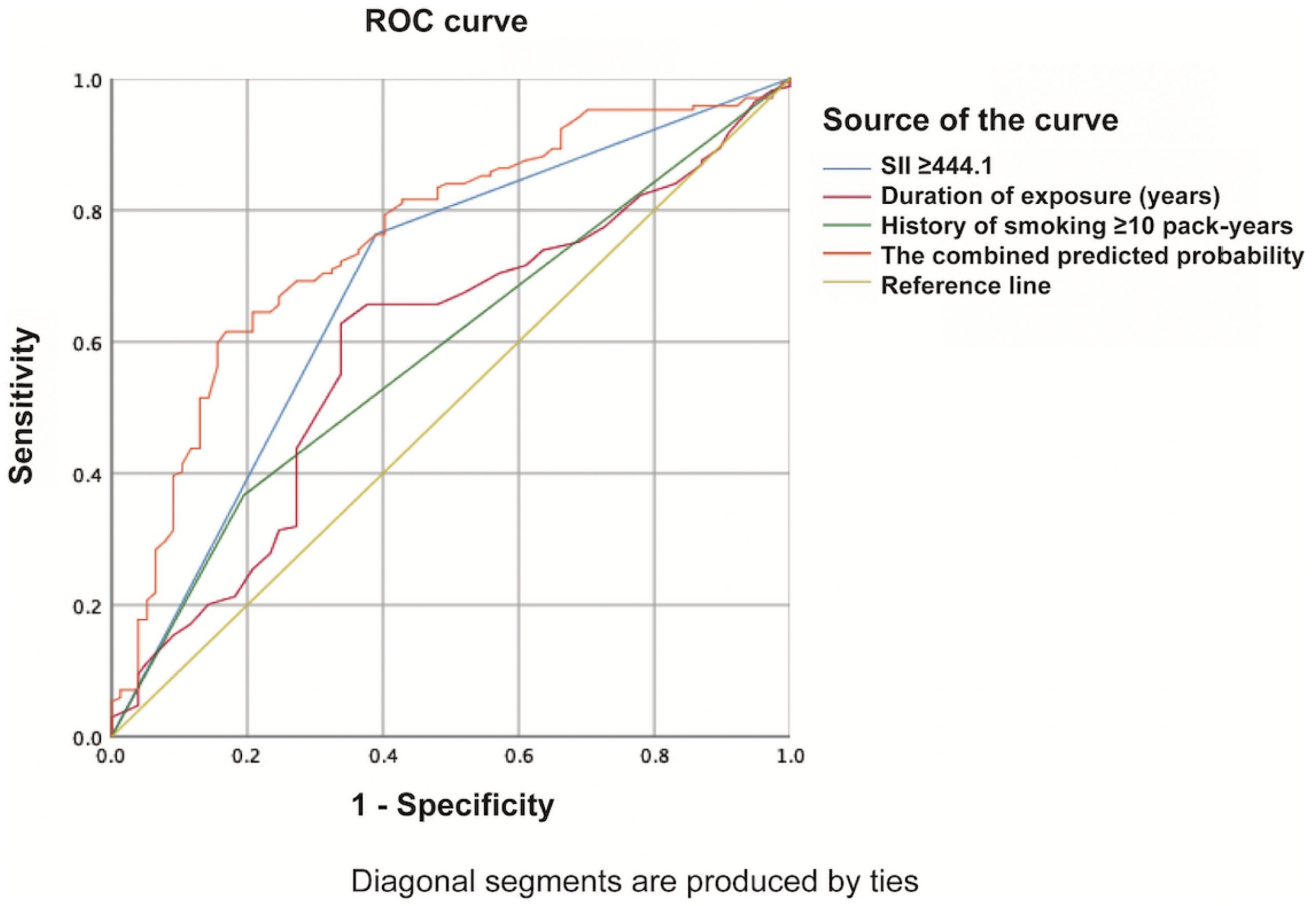
ROC curve of the combination of high SII levels with the other two variables evaluating the severity of silicosis.

The ROC curve was used to evaluate the sensitivity and specificity of high SII levels to predict the advanced stages of silicosis. Although we found that high SII levels had a higher area under a receiver operating characteristic curve (AUC) for identifying more severe silicosis than a history of smoking ≥10 pack-years and duration of silica exposure, we still thought that a single factor could not be sufficient for stratification of the severity of silicosis. Thus, we further established new ROC curves based on the combined markers. As shown in Figure 3, the ROC analysis demonstrated that the combination of high SII levels with the other two variables had the highest AUC of 0.757 (95% confidence interval 0.692–0.822; *p* < 0.001) for identifying the more severe silicosis.

## Discussion

To our knowledge, this is the first study to demonstrate the association between the SII and the severity of silicosis. We found that the patients with advanced stages of silicosis (stage II-III silicosis) had a higher level of SII than the patients with early stages of silicosis (stage I silicosis). Our findings revealed that high SII levels significantly correlated with the advanced stage of silicosis, even after adjusting for various covariates among the patients with silicosis.

Inflammation is a driving factor in the development of silicosis (12). The potential mechanism is that macrophage necrosis after macrophage phagocytosis of silica particles drives inflammation (1). More macrophages again phagocytose the released silica dust, and the repeated process leads to persistent inflammation, thereby contributing to fibrosis (1). Many studies have revealed the relationship between immunity and silicosis. Recently, a multi-omics study based on the silicosis mouse model has emphasized the importance of immune cell chemotaxis, an essential biological process during silicosis development (15). Moreover, peripheral blood T-cell dysregulation has been shown in silica-exposed workers (16). Studies on mouse models have also suggested that T-lymphocyte subsets play an important role in promoting the progression of silicosis (17, 18). Furthermore, increased neutrophil infiltration has been found in the silicosis mouse model (19). Another study showed that mitoDNA could activate neutrophils via TLR9 and cause severe inflammatory responses in the lung tissues of mice with silicosis (20). Although there are few studies about platelets in silicosis, platelet-activation factor (PAF) concentrations have been found to be significantly higher in the plasma of silicosis patients (21). Furthermore, the antagonist of PAF has been shown to improve silica-induced pulmonary fibrosis in animal models (22). Overall, these studies have suggested the close correlation of neutrophils, platelets, and lymphocytes with the pathogenesis of silicosis.

Several studies have explored the correlation of some inflammatory markers obtained from routine blood tests, such as neutrophil-to-lymphocyte ratio (NLR) and platelet-to-lymphocyte ratio (PLR), with silicosis. The NLR and PLR have been found to be higher in patients with silicosis than in unexposed controls and patients exposed to silica without silicosis (23). Studies also indicated their potential value in predicting the prognosis of patients with silicosis (24, 25). In special populations with silicosis, such as engineered stone silicosis patients, these indicators could help to evaluate the disease progression (26). As a new marker, the SII can comprehensively integrate the association between neutrophils, platelets, and lymphocytes, compared with NLR and PLR. Some studies have explored its relation to fibrosis-related diseases, such as interstitial lung disease secondary to connective tissue diseases (CTD-ILD) (11) and liver fibrosis (27). Silicosis is also a fibrotic lung disease (28). Our study explored the relationship between the SII, a novel marker, and silicosis for the first time. The results suggested that higher SII levels, especially with SII values greater than 444.1, were significantly associated with more severe silicosis, even after adjusting for many covariates. However, it is worth noting that the clinical application of the SII is somewhat limited by the fact that blood-based inflammatory indexes are not specific, as they may elevate with respiratory infection or other inflammatory conditions. In addition, tuberculosis, a common complication of silicosis (29, 30), also may potentially alter the inflammatory markers. Therefore, in order to avoid the nonspecific effects of SII, we set strict inclusion and exclusion criteria for the patients’ enrollment, excluding pneumonia, tuberculosis, other infectious diseases, and any condition that could affect hematologic indexes, so as to make the results of the association between SII levels and the severity of silicosis more objective.

Predicting the severity of silicosis by only one factor is of limited value. Our study showed that in addition to higher SII, the duration of silica exposure and a history of smoking ≥10 pack-years were associated with the advanced stages of silicosis. It is well-known that the duration of silica exposure is an important factor in the development of silicosis (1, 5) because exposure duration reflects, to some extent, the cumulative dose of silica (1). As for smoking history, recent animal experimental data have shown that smoking can aggravate inflammation and pulmonary fibrosis of silicosis (31, 32). The findings from these animal studies suggest that cigarette smoking may be associated with the severity of silicosis, thereby supporting our study results. Previous studies have shown an association between smoking and the risk of idiopathic pulmonary fibrosis (33). The proteomic approach also has revealed that smoking exposure leads to a significant dysregulation of many molecular pathways related to interstitial lung diseases (34). As for silicosis, some studies also suggested that cigarette smoking could increase the risk of death in individuals with silica dust exposure (35, 36). This further suggests the harm of smoking, emphasizing the importance of quitting smoking. In addition, our study showed that there were more male patients in the advanced-stage silicosis group, possibly because adult males have more potential for occupational exposure as the primary workforce. Our findings further suggest that the combined indicators, including a higher SII level (SII ≥ 444.1), longer duration of silica exposure, and heavy smoking history, may be better suited for identifying more severe silicosis. This study examined the value of the SII in predicting disease severity, and the diagnostic value of the SII should be further investigated in the future. The diagnostic accuracy of the SII needs to be compared with that of chest radiography-based diagnosis of silicosis to help explore the early and quick diagnosis marker of silicosis.

Previous studies have indicated that lung function impairment increases with silicosis progression (1).

Our study similarly showed impaired pulmonary function in the advanced stage of silicosis relative to the early stage. Lung function in patients with silicosis might present with obstructive, restrictive, or even mixed ventilatory disorders (37–39). The obstructive disorder may be related to the direct inflammatory stimulation of airways induced by inhaled silica particles (40). Restrictive ventilatory disorder may be associated with the progression of interstitial fibrosis (41). As for inflammatory markers, such as NLR and PLR, their correlations with lung function parameters are controversial. A previous study revealed a negative correlation of NLR and PLR with FVC and of NLR with FEV1/FVC (24). However, a recent study has only found an association of NLR with DLCO (23). In contrast, our results showed a significant negative correlation, although weak, between the SII and lung function parameters, such as FVC (% pred), FEV1 (% pred), FEV1/FVC %, and DLCO (% pred). However, correlation does not necessarily mean causation. We rather think that the association was more due to the higher SII values in the more severe stages of silicosis. This finding again suggests that the level of SII might be indicative of more severe stages of silicosis with lung function progression. More importantly, as higher SII levels were related to the advanced stage of silicosis, these patients should be managed and followed up regularly to prevent the development of lung function impairment.

There were still several limitations to the study. First, given the nature of single-center studies, the results may have selection bias. To reduce selection bias to some extent, strict inclusion and exclusion criteria and rigorous study methodology were developed to ensure that the patients included in the study and the results were representative. Second, due to the limited data in the database, more clinical records were lacking. Third, given the limited sample size, there is currently no significant difference between the SII levels of stage II and III silicosis (this part of the data is not presented); the sample size will be expanded in the future to explore the subgroups of different stages for more detailed analyses, making the results more convincing. Therefore, prospective large-scale multicenter studies will need to confirm our findings in the future.

## Conclusion

In summary, our findings showed that higher SII levels are associated with more severe silicosis, and the best cutoff value of SII is 441.1. There is a significant but weak negative correlation between SII levels and lung function parameters. The SII, a comprehensive and easily accessible potential biomarker, might be used to help identify the severity of silicosis routinely. However, given the non-specificity of the inflammatory markers in general and the limitation of the single-center retrospective study, more long-term, large-scale studies are required to validate these findings.

## Data availability statement

The original contributions presented in the study are included in the article/Supplementary material, further inquiries can be directed to the corresponding author.

## Ethics statement

The studies involving humans were approved by The Research Ethics Board of Beijing Chao-Yang Hospital (project approval number: 2023-ke-357). The studies were conducted in accordance with the local legislation and institutional requirements. Written informed consent for participation was not required from the participants or the participants’ legal guardians/next of kin in accordance with the national legislation and institutional requirements.

## Author contributions

H-Y-JK: Conceptualization, Data curation, Methodology, Writing – original draft. S-YC: Data curation, Methodology, Writing – original draft. SS: Data curation, Writing – original draft. L-RL: Methodology, Writing – review & editing. Z-HT: Conceptualization, Funding acquisition, Writing – review & editing.
